# Isolated Aspergillosis Myocardial Abscesses in a Liver-Transplant Patient

**DOI:** 10.1155/2014/418357

**Published:** 2014-02-23

**Authors:** Kim-Diêp Dang-Tran, Valérie Chabbert, Laure Esposito, Céline Guilbeau-Frugier, Fabrice Dédouit, Lionel Rostaing, Hervé Rousseau, Phillippe Otal, Nassim Kamar

**Affiliations:** ^1^Service de Radiologie Générale, Centre Hospitalier Universitaire Rangueil, 1 avenue du Professeur Jean Poulhès, TSA 50032, 31059 Toulouse Cedex 9, France; ^2^Department of Nephrology and Organ Transplantation, CHU Rangueil, TSA 50032, 31059 Toulouse Cedex 9, France; ^3^Service d'Anatomo-Pathologie, Centre Hospitalier Universitaire Rangueil, 1 avenue du Professeur Jean Poulhès, 31059 Toulouse Cedex 4, France; ^4^Université Paul Sabatier, 31059 Toulouse, France; ^5^Service de Médecine Légale, Centre Hospitalier Universitaire Rangueil, 1 avenue du Professeur Jean Poulhès, 31059 Toulouse Cedex 4, France; ^6^INSERM U1043, IFR-BMT, CHU Purpan, 31059 Toulouse, France

## Abstract

Cardiac abscess is an uncommon and fatal complication after transplantation. We report a case of an initially isolated aspergillosis myocardial abscess diagnosed by cardiac magnetic resonance imaging (CMRI). At that time, there was no other biological evidence or other extracardiac manifestations. A three-month course of dual antifungal therapy followed by a single antifungal therapy was empirically given. Six month after admission, *Aspergillus fumigatus* was isolated for the first time and the patient deceased from a disseminated aspergillosis.

## 1. Introduction

Infections are commonly observed after solid-organ transplantation and generally require rapid and adequate therapy within this setting [1]. Although they seldom occur in cardiac locations, endocarditis is the main heart infection observed, whereas a cardiac abscess is rare. Concomitant clinical and/or biological abnormalities usually lead to etiologic diagnosis of a cardiac abscess. Herein, we report a case of an initially isolated cardiac abscess that occurred in a liver-transplant patient. Cardiac magnetic resonance imaging (CMRI) was found to be helpful in assessing the evolution of the abscess under empiric therapy.

## 2. Case Presentation

A 68-year-old man underwent a first cadaveric orthotopic liver transplantation for hepatitis B virus and alcohol-induced cirrhosis that was complicated by hepatocellular carcinoma. After an induction therapy using antithymocyte globulins (ATG), his initial immunosuppressive therapy was based on tacrolimus, mycophenolate mofetil, and steroids. At day 11, because *Aspergillus fumigatus* was found in a systematic tracheal aspiration, a 21-day course of voriconazole was given. By day 60, he experienced a steroid-resistant acute rejection, which required ATG antirejection therapy. By day 78, he presented with isolated fever. C-reactive protein was 100 mg/L. Total lymphocyte count was 375/mm^3^. CD4-positive cell count was 60/mm^3^. Liver function tests were within the normal ranges. Blood-culture and urine-culture were negative for bacteria and fungi. *Aspergillus* antigenemia was negative. *Cytomegalovirus* DNAemia was negative. The bronchoalveolar lavage did not reveal the presence of any bacteria, virus, or fungi. In the absence of any identified cause of infection, he underwent a chest and abdominal CT scan, which showed an isolated rounded cardiac collection. Thus, a cardiac MRI was performed as soon as possible to enable diagnosis and close monitoring of the evolution of the cardiac lesion.

The CMRI pointed out a 3 cm diameter collection within the epicardium and myocardium, on the apex posterior wall, without any mass effect. On T1-weighted MRI, the lesion showed a thick rim and a hypointense core. The core was hyperintense on T2-weighted and T2-black blood SPIR MRI, suggesting a cystic core. Only the thick rim was enhanced on postgadolinium MRI. No ischemic lesion or heart-motion abnormality was found. Thus, we hypothesized an infectious cardiac abscess. Because the abscess was very close to the anterior interventricular artery, the radiologists and cardiac surgeons could not perform any abscess puncture for microbial diagnosis. Despite a negative *Aspergillus* antigenemia and because of the patient's recent history of aspergillosis, we initiated a dual antifungal therapy of caspofungin and liposomal Fungizone.

**Figure 1:**
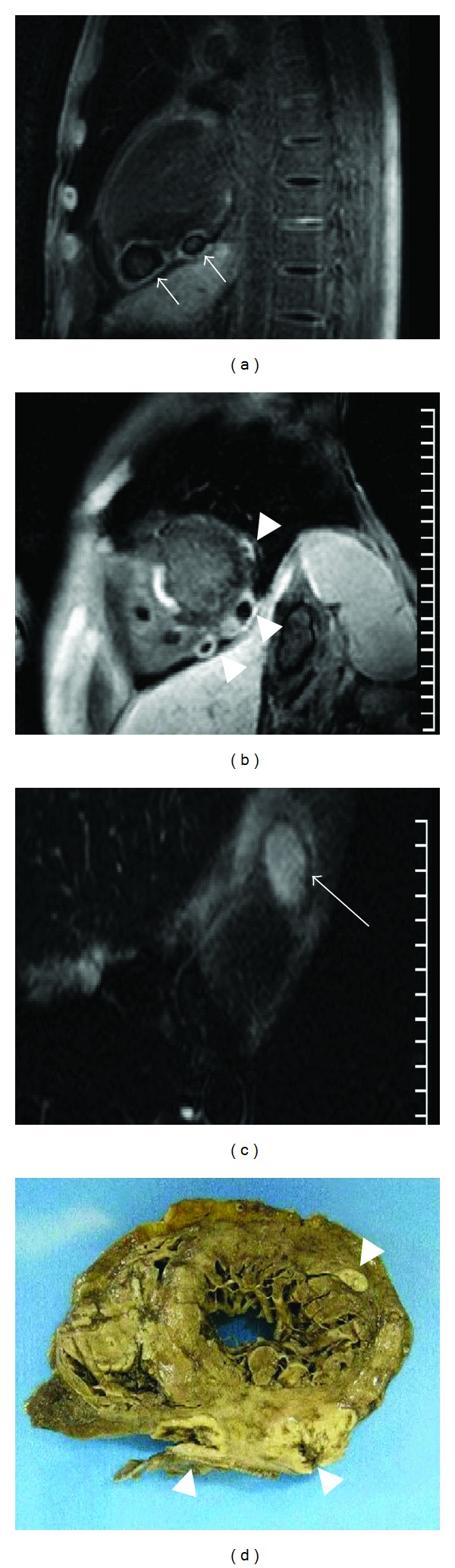
Cardiac MRI performed 6 months after diagnosis and autopsy study. (a) T1-weighted postgadolinium MRI—long axis view. Two collections (white arrows) with thick enhancing rim and hypointense core in the epicardium and myocardium of the left ventricle posterior wall. (b) T1-weighted postgadolinium MRI—short axis view. Lesions (arrow heads) in the left ventricle wall. A hyperintense calcified lesion located in the interventricular septum is also visualized. (c) T2-black blood MRI—axial view. Hyperintense core suggesting a cystic core (black arrow). (d) Autopsy study. Heart short axis view pointing out the three left ventricle abscesses observed on image (b) (arrow heads).

A second CMRI, performed one week later, showed the abscess had grown. Because of a history of tuberculosis in childhood, this prompted us to initiate an antituberculosis therapy (isoniazid, rifampicin, ethambutol, and pyrazinamide) though mainly pericardial abscess has been described [2]. A CMRI performed 3 months after diagnosis showed stability of the abscess, with the patient still receiving antituberculosis and double antifungal therapies. At that time, liposomal Fungizone was replaced by voriconazole. Because of increased liver-enzyme levels, voriconazole was stopped 1 month later, and the patient was continued on caspofungin alone. Antituberculosis therapy was also continued. By 6 months after diagnosis, a CMRI showed several new growing epi- and myocardial abscesses on both left and right atria and ventricles (Figure 1). Multiple small and confluent soft-contoured nodules were observed on CT examination and could definitively be attributed to *Aspergillus fumigatus* infection as, at that time point, the latter was found for the first time in serum, blood, urine, and lung. For the first time, *Aspergillus* antigens were also positive. Urgent heart surgery was discussed but was rejected due to the patient's altered physiological status and to the extent of the lesions. A few days later, the patient died from a septic shock and multiple organ failure caused by disseminated aspergillosis. Postmortem examination confirmed that multiple aspergillosis abscesses were located in the epicardium, myocardium, and endocardium and were associated with lung, liver, kidney, and skin aspergillosis abscesses.

## 3. Discussion 

Pulmonary form of invasive aspergillosis is commonly observed after transplantation, but it is seldom located on the heart wall. Few aspergillosis cardiac locations have been described in severely immunocompromised AIDS patients with less than 200 CD4 T-lymphocytes/*μ*L [3], but no cardiac abscess has been related after solid transplantation. To the best of our knowledge, this is the first reported case of cardiac aspergillosis abscess as first aspergillosis imaging abnormality in a transplanted patient. At admission there was no evidence of aspergillosis. *Aspergillus* antigenemia remained negative until 6 months after the diagnosis of the myocardial abscess. *Aspergillus* was also not found in the BAL. The antifungal therapy was initiated based on the patient's posttransplant history, that is, heavy immunosuppression and *Aspergillus fumigatus* detected in tracheal aspiration taken early after transplantation. Under dual antifungal therapy, the abscess size remained stable. Three months after the presentation, one of the two antifungal drugs was withdrawn: a disseminated aspergillosis was observed. However, the relationship between the number of antifungal drugs used and the outcome cannot be established.

Other organisms are known to cause a myocardial abscess: *Staphylococcus aureus, Streptococcus*, *Candida*, and *Salmonella *[4]. Those myocardial abscesses have been described with quite similar imaging characteristics but can be diagnosed with specific biological assessments (e.g., for *Staphylococcus aureus*, blood cultures) [5]. Our patient did not show any positive results from these assessments.

In the present case, CMRI was a valuable asset and permitted us to detect heart-wall lesions and to assess their extent and progression and enabled a definitive diagnosis: a collection with a thick enhancing rim. CMRI also allowed us to study the remaining heart-wall thickness and to evaluate the rupture prognosis and heart-wall motion, which is in contrast to results obtained using CT. With its precise images, CMRI could offer guidance to a surgical approach. The use of CMRI is an efficient, simple, accurate, and noninvasive technique but does lack etiology specificity. Other methods to detect a cardiac abscess have their shortfalls. Transthoracic echocardiography, performed a few days after the first CMRI, was only able to show an inferior pericardial thickening, due to reduced apex visibility. A transoesophageal echocardiography could not be planned because of the patient's altered clinical status. At admission, a puncutre of the abcess may have allow the early identification of the *Aspergillus*, and to adapt anti-fungal therapy consequently.

In conclusion, myocardial abscess can be observed as first imaging evidence of an invasive aspergillosis. Early diagnosis and treatment are required because of the high risk of mortality.
